# Safety of 4-factor prothrombin complex concentrate (4F-PCC) for emergent reversal of factor Xa inhibitors

**DOI:** 10.1186/s40560-018-0303-y

**Published:** 2018-06-14

**Authors:** Jing Tao, Elena N. Bukanova, Shamsuddin Akhtar

**Affiliations:** Department of Anesthesiology, Yale School of Medicine, 333 Cedar Street, TMP 3, PO Box 20805, New Haven, CT 06520-8051 USA

**Keywords:** 4-factor prothrombin complex concentrates, Novel oral anticoagulant, Factor Xa inhibitor, Anticoagulation reversal, Thromboembolism, Drug safety

## Abstract

**Background:**

Although factor Xa inhibitors have become a popular choice for chronic oral anticoagulation, effective drug reversal remains difficult due to a lack of specific antidote. Currently, 4-factor prothrombin complex concentrate (4F-PCC) is considered the treatment of choice for factor Xa inhibitor-related major bleeding. However, safety of 4F-PCC and its risk of thrombosis when used for this off-label purpose remain unclear. The purpose of this retrospective study is to determine the rate of thromboembolism when 4F-PCC is used for the emergent reversal of factor Xa inhibitors.

**Methods:**

We conducted a single-center retrospective review of medical records between 2013 and 2017. Patients were included if they received 4F-PCC to reverse rivaroxaban, apixaban, or edoxaban for emergent invasive procedures or during episodes of major bleeding defined as bleeding with hemodynamic instability, fall in hemoglobin of 2 g/dL, or bleeding requiring blood transfusion. Thrombotic events including myocardial infarction, pulmonary embolism, deep vein thrombosis, cerebral vascular accident, and arterial thrombosis of the limb or mesentery were recorded if they occurred within 14 days of 4F-PCC administration. Data was analyzed using point and interval estimation to approximate the rate and confidence interval of thromboembolic events.

**Results:**

Forty-three patients were identified in our review. Doses of 4F-PCC were determined by the treating physician and mainly ranged from 25 to 50 IU/kg. Twenty-two patients (51.2%) received both sequential compression devices (SCDs) and subcutaneous heparin for DVT prophylaxis. Twenty-one patients (48.8%) were placed on SCDs only. Three patients received concomitant FFP. Thrombotic events within 14 days of 4F-PCC administration occurred in 1 out of 43 patients (2.1%, 95% CI [0.1–12.3]). This thrombotic event was an upper extremity DVT which occurred 1 day after the patient received 1325 IU (25 IU/kg) of 4F-PCC to reverse rivaroxaban for traumatic intracranial hemorrhage. The patient was taken for emergent decompressive craniotomy after rivaroxaban reversal. In patients who did not undergo surgery or who underwent minor invasive procedures, no thrombotic events were noted.

**Conclusion:**

Based on our preliminary data, the thromboembolic rate of 4F-PCC when given at a dose of 25–50 IU/kg to emergently reverse rivaroxaban and apixaban appears acceptable. Since many patients who require 4F-PCC to emergently reverse factor Xa inhibitors will be at high risk of developing thrombotic events, practitioners should be highly vigilant of these complications. Large, multicenter prospective trials are needed to further determine this risk.

## Background

For over five decades, warfarin has been the preferred treatment for patients requiring long-term oral anticoagulation. In 2010, dabigatran, a direct thrombin inhibitor and the first non-vitamin K antagonist oral anticoagulant, was approved by the FDA for stroke prevention in patients with non-valvular atrial fibrillation [1]. The first factor Xa inhibitor, rivaroxaban, was approved for stroke prevention, treatment of deep vein thrombosis (DVT), and pulmonary embolism (PE) in 2011 [2]. Together, these direct oral anticoagulants (DOACs), which now include apixaban and edoxaban, have increasingly become the drug of choice for patients requiring chronic anticoagulation.

DOACs possess several advantages over warfarin, including more predicable pharmacokinetics, minimal food and drug interactions, and lack of blood draws for drug monitoring [3]. In addition, rivaroxaban was found to have lower rates of both intracranial and fatal bleeding, while apixaban proved more effective in stroke prevention and lowered mortality [4, 5].

Despite these benefits, DOAC reversal has proven difficult. In 2015, idarucizumab was approved by the FDA for the reversal of direct thrombin inhibitor dabigatran. However, reversal of factor Xa inhibitors remains challenging due a lack of specific antidote. The use of fresh frozen plasma (FFP) has so far not been studied. Current expert opinion recommends against its use as it may not be sufficient to overcome the effect of the anticoagulant drug [6–8]. Recombinant factor VIIa (rFVIIa) has been effective in some small studies using animals and humans, but results are conflicting [9–13]. Four-factor prothrombin complex concentrate (4F-PCC), although not FDA approved for factor Xa inhibitor reversal, appears to show the most promise. In animals treated with factor Xa inhibitors, 4F-PCC improved coagulation profile, decreased blood loss, and normalized bleeding time [9, 10]. In humans, data is more limited, but also supports the use of 4F-PCC. In a randomized trial by Eerenberg et al., 4F-PCC corrected endogenous thrombin potential and prothrombin time in healthy volunteers treated with a single dose of rivaroxaban [14]. Similarly, Schultz el al. found that 4F-PCC improved thrombin generation and clotting time when given to patients on chronic rivaroxaban therapy [13].

4F-PCC safety, unlike its efficacy, has so far only been studied in vitamin K antagonist reversal. Given the high levels of coagulation factors contained in 4F-PCC and the underlying pro-thrombotic conditions for which DOACs are typically prescribed, the thrombotic risk associated with 4F-PCC use is of obvious concern. The purpose of this pilot study is to determine the rate of thromboembolic events in patients who received 4F-PCC for the reversal of factor Xa inhibitors during episodes of major bleeding or need of emergent surgery or invasive procedure.

## Methods

### Study design

We conducted a retrospective review of medical records between January 2013 and May 2017 at Yale-New Haven Hospital, a tertiary care hospital and level 1 trauma center. The project received approval from the hospital’s Institutional Review Board, and patients were identified using an inpatient pharmacy database. Patients were included in this review if they received 4F-PCC for the purpose of reversing rivaroxaban, apixaban, or edoxaban for emergency surgery or invasive procedures, or during episodes of major bleeding defined as bleeding with hemodynamic instability, fall in hemoglobin of 2 g/dL, or bleeding requiring blood transfusion. Patients were excluded if they received 4F-PCC for purposes other than reversing factor Xa inhibitors or if they were under 18 years of age. 4F-PCC used at our institution is Kcentra® (CSL, Bering).

Two anesthesiologists independently completed chart reviews of included patients from an electronic medical record (EMR) using a standardized data abstraction form. Parameters extracted from each patient’s EMR included age, gender, weight, past medical history, indication for oral anticoagulation, dose of anticoagulant prior to admission, cause of major bleeding or reason for anticoagulation reversal, time and dose of 4F-PCC administered, surgical or procedural interventions performed, and deep vein thrombosis prophylaxis used during admission. Thrombotic events including acute deep vein thrombosis, pulmonary embolism, myocardial infarction or acute coronary syndrome, transient ischemic stroke, cerebral vascular accidents, and arterial thrombosis of limb or mesentery were also recorded.

Previous studies on the efficacy of 4F-PCC for oral anticoagulation reversal have used various time periods to report thrombotic events related to 4F-PCC, ranging from 7 days to hospital discharge to death [15–18]. Rationale for using a specific time frame was not mentioned in these studies.

In our study, we considered thrombotic events to be a potential complication of 4F-PCC if they occurred within 14 days of drug administration. We chose 14 days based on pharmacokinetics of the coagulation factors contained in 4F-PCC. After single administration, first pass pharmacokinetics dictate that 50% of a drug will remain after one half-life, 25% after two half-lives, 12.5% after three half-lives, 6.25% after four half-lives, and 3.125% after five half-lives. Coagulation factors contained within 4F-PCC have various half-lives, with factor II having the longest half-life of 60 h [19]. Factor II level is also thought to be highly associated with thrombotic events [20, 21]. Per first order pharmacokinetics, after 336 h or 14 days, exogenous factor II should theoretically have undergone five half-lives elimination and be minimally present in plasma. Thrombotic events occurring after this period would therefore be unlikely the result of 4F-PCC and more likely due to other factors such as underlying medical conditions, immobility, or lack of thrombotic prophylaxis.

Data were de-identified and stored in a simple database. To ensure fidelity of data abstraction, all of the included charts were analyzed by both reviewers, with inter-observer agreement found to be congruent on all data fields.

### Statistical analysis

Data was analyzed using point and interval estimation to approximate the rate and confidence interval of thromboembolic events in the general population from our sample size. A breakdown of this study’s thrombotic events was reported in a descriptive fashion.

## Results

A total of 43 patients received 4F-PCC for emergent rivaroxaban or apixaban reversal at our institution between January 2013 and May 2017. We did not find any subjects who required emergent reversal of edoxaban. An additional 21 patients were also identified as having received 4F-PCC for factor Xa inhibitor reversal. However, these patients received a single non-weight-based dose of 50 IU. Because the recommended dose of 4F-PCC for factor Xa inhibitor reversal is 50 IU/kg, a dose of 50 IU would be considered extremely low and not likely contribute to thrombotic development [22]. As a result, we did not include these patients in our analysis. Subject demographics, medical history, and clinical events are summarized in Table 1.

**Table 1:** Demographics

Demographics	*n* (%)
Total patients	43
Median age (year)	74
Gender	
Male	23 (53.5)
Female	20 (46.5)
Anticoagulation	
Rivaroxaban	21 (48.8)
Apixaban	22 (51.2)
Edoxaban	0
Indication for anticoagulation	
Atrial fibrillation	30 (69.8)
DVT/PE	9 (20.9)
Atrial fibrillation and DVT/PE	3 (7.0)
Lower extremity venous bypass graft	1 (2.3)
Indication for anticoagulation reversal	
Gastrointestinal bleeding	17 (39.5)
Intracranial hemorrhage—non-traumatic	9 (20.9)
Intracranial hemorrhage—traumatic	7 (16.3)
Trauma	5 (14.0)
Other	5 (11.6)
Invasive procedure after 4F-PCC administration	30 (69.8)
Gastrointestinal endoscopies	16 (37.2)
Interventional radiology embolization/coiling	3 (7.0)
Craniotomy	3 (7.0)
Orthopedic	3 (7.0)
Other	5 (11.6)

The median age at time of 4F-PCC administration was 74 (range 20–94). 53.5% of the patients were men. The indications for anticoagulation with factor Xa inhibitors were as follows: history of atrial fibrillation (*n* = 30, 69.8%), DVT/PE (*n* = 9, 20.9%), both atrial fibrillation and DVT/PE (*n* = 3, 7%), and lower extremity venous bypass graft (*n* = 1, 2.3%). Twenty-two of the 43 patients (51.2%) were on apixaban therapy at the time of 4F-PCC administration. Twenty-one patients (48.8%) were on rivaroxaban. Conditions requiring anticoagulation reversal were mainly gastrointestinal (GI) bleeding (*n* = 17, 39.5%), non-traumatic intracranial hemorrhage (*n* = 9, 20.9%), traumatic intracranial hemorrhage (*n* = 7, 16.3%), and traumatic injuries (*n* = 5, 14.0%). Thirty of the 43 patients (69.8%) underwent an invasive procedure after receiving 4F-PCC. The most common invasive procedures were GI endoscopies, including both esophagogastroduodenoscopy (EGD) and colonoscopy, performed in 16 patients (37.2%).

Doses of 4F-PCC used to reverse factor Xa inhibitors were variable and chosen at the discretion of the treating physician (Table 2). Twenty-two of the 43 patients (51.2%) received 25 IU/kg, 4 patients (9.3%) received 25–50 IU/kg, 16 patients (37.2%) received 50 IU/kg, and 1 patient (2.3%) received over 50 IU/kg. The rationale behind the dose selection was not apparent through our review of medical records. Three patients (6.9%) received concurrent FFP.

**Table 2:** Dose of 4F-PCC used for factor Xa inhibitor reversal

Dose of 4F-PCC	*n* (%)
25 U/kg	22 (51.2)
25–50 U/kg	4 (9.3)
50 U/kg	16 (37.2)
> 50 U/kg	1 (2.3)

The hemostatic efficacy of 4F-PCC was determined by the treating physician based on clinical measures. These included patient hemodynamics, trend of hemoglobin and hematocrit, and active bleeding as seen on imaging or invasive procedures. Of the 43 patients in the study, only three patients (6.9%) continued to have active bleeding after receiving 4F-PCC. Two of these three patients subsequently died as a result of hemorrhage, while one required surgery to achieve hemostasis.

Coagulation tests were obtained for all 43 patients prior to 4F-PCC administration. Ten patients (23.3%) had elevated prothrombin time (PT) and international normalizing ratio (INR) when the hemorrhage occurred. Five (50%) of these patients were on rivaroxaban and five (50%) on apixaban. PT/INR returned to normal limits after 4F-PCC administration in six of the ten patients (60%). PT/INR improved but not to normal limits in two of the ten patients (20%). Two patients (20%) did not have post-reversal PT/INR drawn. Factor Xa levels were not drawn for any patients in our study. At the time of this retrospective review, 21 of the 43 patients (48.8%) were still alive.

Timing and type of DVT prophylaxis used during the hospital admission also varied (Table 3). Twenty-one patients (48.8%) were given only sequential compression devices (SCDs) for DVT prophylaxis. Twenty-two of the 43 patients (51.2%) received both subcutaneous heparin and SCDs for DVT prophylaxis during hospital admission. Of these 22 patients, 12 patients (27.9%) were started on subcutaneous heparin within 48 h of 4F-PCC administration.

**Table 3:** Timing and type of DVT prophylaxis after 4F-PCC administration

DVT prophylaxis	*n* (%)
Sequential compression device only	21 (48.8)
Subcutaneous heparin and sequential compression device	22 (51.2)
Subcutaneous heparin started within 48 h of 4F-PCC administration	12 (27.9)
Subcutaneous heparin started after 48 h of 4F-PCC administration	10 (23.3)

Thromboembolic events within 14 days of receiving 4F-PCC occurred in 1 out of 43 patients (2.1%, 95% CI [0.1–12.3]) (Table 4). The thrombotic event was an acute upper extremity DVT which occurred in a 20-year-old male who received 4F-PCC to reverse rivaroxaban in the setting of intracranial hemorrhage. The patient was admitted for headache after sustaining a mechanical fall. Subsequent radiographic imaging showed a large intra-parenchymal hemorrhage with midline shift. His mental status at that time was somnolent but appropriate. One thousand three hundred twenty-five units (25 IU/kg) of 4F-PCC was given approximately 4.5 h after his last dose of oral anticoagulation. He was subsequently taken for emergent decompressive craniotomy. The patient’s DVT occurred on post-operative day 1, with the patient on both SCDs and subcutaneous heparin for DVT prophylaxis. He did not receive any concurrent FFP during this event. This patient had been placed on rivaroxaban as an outpatient for history of PE associated with traveling.

**Table 4:** Thromboembolic events within 14 days of 4F-PCC administration

Thromboembolic event	*n* (%)
Total	1 (2.3)
Deep vein thrombosis	1 (2.3)
Pulmonary embolism	0
Myocardial infarction	0
Cerebral vascular accident	0
Arterial thrombosis of limb	0
Arterial thrombosis of mesenteries	0

One other thrombotic event was identified in our chart review. This event was a subsegmental PE which occurred 3 months after the patient received 4F-PCC for apixaban-associated GI bleed. This patient was not restarted on chronic anticoagulation after hospital discharge.

## Discussion

The use of DOACs has significantly increased in recent years because of their favorable pharmacokinetic profile and ease of use. Although idarucizumab has been developed for the reversal of dabigatran, no specific antidote currently exists for factor Xa inhibitors. Two specific Xa inhibitor reversal agents, andexanet alpha and ciraparantag, have shown promise in clinical trials but have yet to gain FDA approval. Currently, 4F-PCC is considered the treatment of choice for factor Xa inhibitor reversal. Animal studies using 4F-PCC to reverse rivaroxaban and apixaban have shown general success in reducing blood loss and bleeding time [9–11]. In humans, no clinical trials currently exist on the efficacy of 4F-PCC during active bleeding associated with factor Xa inhibitors. However, in vitro and ex vivo studies in healthy volunteers treated with factor Xa inhibitors found that 4F-PCC improved endogenous thrombin potential and thrombin concentration [12–14, 23].

Evidence on 4F-PCC dosing for factor Xa inhibitor reversal also remains limited. When used to reverse warfarin, 4F-PCC is dosed by presenting INR. The 25 IU/kg is given for INR 2 to less than 4, 35 IU/kg for INR 4 to 6, and 50 IU/kg for INR over 6 [24, 25]. However, INR as well as active partial prothrombin time (aPTT) does not correlate well with degree of anticoagulant effect induced by factor Xa inhibitors [18, 26–28]. PT may be used to monitor rivaroxaban level, but results are highly variable depending on the reagent used [28, 29].

To determine appropriate dosing of 4F-PCC for factor Xa inhibitor reversal, a variety of doses ranging from 25 to 100 IU/kg has been tested. Perzborn et al. found that 50 IU/kg of 4F-PCC decreased bleeding time in primates treated with rivaroxaban [10]. The authors did not find bleeding time reduction when 25 IU/kg was used. In healthy human volunteers given rivaroxaban, Marlu et al. found that 25 IU/kg of 4F-PCC fully corrected endogenous thrombin potential and partially corrected peak thrombin concentration. The study also found that 50 IU/kg increased thrombin generation back to baseline [12]. Similarly, Escolar et al. found that 50 IU/kg of 4F-PCC improved thrombin generation in healthy human subjects treated with apixaban [23]. The updated European Heart Rhythm Association practical guideline in 2017 recommends using 50 IU/kg if full factor Xa inhibitor reversal is desired [22].

Unlike efficacy and dosing, safety of 4F-PCC use in anticoagulation reversal has only been studied in vitamin K antagonists. When given for warfarin-related bleeding, 4F-PCC has a thrombotic risk ranging from 1.8 to 9.1% [30–33]. The rate of thrombosis when it is used to treat factor Xa inhibitors remains unclear.

Although some studies on 4F-PCC have noted their own thrombotic events as part of safety reporting, ours is the first to specifically examine this risk over time. We found one case of thrombotic complications in our retrospective review of 43 patients, with an overall rate of 2.3%.

The thrombotic event occurred in a patient who carried high risks of developing venous thrombosis in addition to the underlying conditions for which they required chronic anticoagulation. The patient was given 4F-PCC shortly before undergoing emergent decompressive craniotomy for cerebral hemorrhage resulting from a fall. Since trauma and major surgery are both major risk factors for developing venous thrombosis, 4F-PCC may have exacerbated this hypercoagulable state [34].

We found no thrombotic complications in patients who had not undergone major surgery or who underwent minor invasive procedures such as GI endoscopies.

We also found one other thrombotic event which occurred 3 months after 4F-PCC administration. We do not believe this event was related to 4F-PCC administration since the drug would have been completely metabolized by that time. In addition, the patient who suffered this late thrombotic event was also not restarted on chronic anticoagulation therapy after hospital discharge.

Although the observed thrombotic rate of 2.3% in our patients is notable, we believe it is an understandable risk given the potential contributing factors. First, medical DVT prophylaxis with subcutaneous heparin was not frequently used in our patients after 4F-PCC administration, likely out of concern for further bleeding. Only half our study patients received subcutaneous heparin during their hospital admission. The other half were only treated with SCDs. Second, patients who are required to be on chronic anticoagulation, such as those with a history of atrial fibrillation and DVT, will have an inherently higher risk of thrombosis due to their underlying medical condition.

Our study has several limitations. First, this is a single-center retrospective chart review with a small sample size. Although we reviewed data from the past 5 years, 4F-PCC has only been recently recommended for DOAC reversal [22, 35]. This is apparent in our study as 74% of 4F-PCC administration occurred in the last 2 years of the review period. Second, thrombotic events other than DVT and PE may not have been detected given a small sample size from one institution. As such, the results of this pilot study may not reflect the experiences of other medical centers. Third, a large number of patients in our review received 4F-PCC at a lower dose than what is recommended for full factor Xa inhibitor reversal. 51.2% of our study patients received 25 IU/kg while 9.3% received 25–50 IU/kg. Only 37.2% of patients received the recommended 50 IU/kg. Although 25 IU/kg has been shown to provide incomplete reversal of factor Xa inhibitors and would be a reasonable starting dose in patients with extremely high risk of thrombosis, this under-dosing may have contributed to our observed low rate of thromboembolic events [12, 22].

## Conclusion

Based on our preliminary data, the thrombotic rate of 4F-PCC when given at a dose of 25–50 IU/kg to emergently reverse rivaroxaban and apixaban appears acceptable. Because many patients who require 4F-PCC for emergent factor Xa inhibitor reversal will be at high risk of developing thrombosis, practitioners should be highly vigilant of these complications, especially in the immediate period following 4F-PCC administration. Large, multicenter prospective studies are needed to further determine this risk.

## Abbreviations

4F-PCC: 4-factor prothrombin complex concentrate
aPTT: Activated partial prothrombin time
DOAC: Direct oral anticoagulant
DVT: Deep vein thrombosis
EGD: Esophagogastroduodenoscopy
EMR: Electronic medical records
GI: Gastrointestinal
INR: International normalized ratio
PCC: Prothrombin complex concentrate
PE: Pulmonary embolism
PT: Prothrombin time
rFVIIa: Recombinant factor VIIa
SCD: Sequential compression device
